# MS and NMR Analysis of Isotopically Labeled Chloramination Disinfection Byproducts: Hyperlinks and Chemical Reactions

**DOI:** 10.1021/acs.analchem.3c03888

**Published:** 2024-05-09

**Authors:** Justinas Sakas, Ezra Kitson, Nicholle G. A. Bell, Dušan Uhrín

**Affiliations:** EaStCHEM School of Chemistry, University of Edinburgh, David Brewster Rd, Edinburgh EH9 3FJ, U.K.

## Abstract

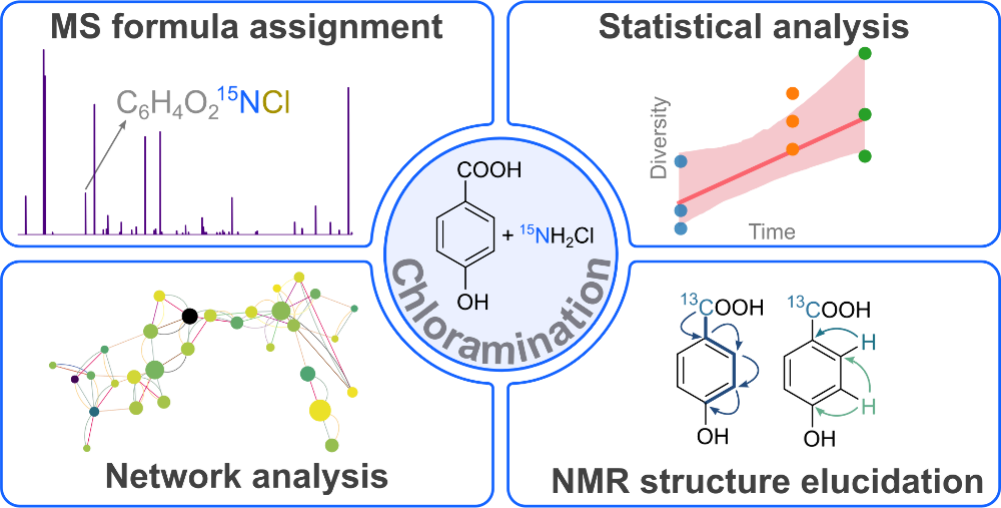

FT-ICR MS and NMR analysis of an isotopically labeled complex mixture of water disinfection byproducts formed by chloramine disinfection of model phenolic acids is described. A new molecular formula assignment procedure using the CoreMS Python library able to assign isotopically enriched formulas is proposed. Statistical analysis of the assigned formulas showed that the number of compounds, the diversity of the mixture, and the chlorine count increase during the chloramination reaction. The complex reaction mixture was investigated as a network of reactions using PageRank and Reverse PageRank algorithms. Independent of the MS signal intensities, the PageRank algorithm calculates the formulas with the highest probability at convergence of the reaction; these were chlorinated and nitrated derivatives of the starting materials. The Reverse PageRank revealed that the most probable chemical transformations in the complex mixture were chlorination and decarboxylation. These agree with the data obtained from INADEQUATE NMR spectra and literature data, indicating that this approach could be applied to gain insight into reactions pathways taking place in complex mixtures without any prior knowledge.

## Introduction

The analysis and structure elucidation of complex mixtures is an arduous task as complex mixtures contain hundreds to thousands of compounds that cannot be separated using chromatographic techniques.^1^ Fourier-transform ion cyclotron resonance mass spectrometry (FT-ICR MS) offers an extremely low detection limit and high resolution and thus can be used for untargeted analysis of complex mixtures, bypassing the customary chromatographic step. A number of FT-ICR MS methods for complex mixture analysis have been reviewed extensively.^2−4^ Untargeted FT-ICR MS analysis mostly focused on the analysis of natural organic matter,^5−9^ but has also been applied to other complex matrices such as beverages^10−12^ and crude oil.^13−15^

In this paper, we use FT-ICR MS for the analysis of ^15^N-labeled complex mixtures. We apply this method to investigate chloramination disinfection byproducts (DBPs), a complex mixture with real-world relevance. Chloramination is a water disinfection method used around the world to provide safe potable water. The addition of chloramine (NH_2_Cl) kills pathogens; however, chloramine also reacts with the natural dissolved organic matter (DOM) and anthropogenic contaminants to produce a complex mixture of DBPs, which are known to be cytotoxic, genotoxic, and carcinogenic.^16^ Although the levels of some of these compounds are regulated, the majority of byproducts are unregulated and more than 50% of the byproducts have not been chemically identified.^17^ Additionally, it has been shown that the total toxicity of DBPs cannot be accounted by regulated compounds alone,^18,19^ and a recent study reported that it is largely dominated by the unregulated DBPs.^20^

FT-ICR analysis of halogenated DBPs has been performed by multiple groups in recent years^21−26^ and ^15^N-labeling has been previously used to investigate known chloramination DBPs.^27,28^ To our knowledge, this work is the first to combine the two approaches and include isotopic labeling for untargeted FT-ICR MS analysis of mixtures containing halogens.

We have developed a model chloramination reaction using compounds which share structural features with DOM in conjunction with ^15^N-labeled chloramine to perform the disinfection.^29^ Chloramination is known to produce less regulated DBPs compared to chlorination;^30^ however, it produces N-containing compounds which are more toxic and hence of special interest.^31^ The addition of labeled chloramine increases confidence in the assigned formula as it is the only source of ^15^N (as the major isotope) and also opens up avenues for future analysis of nitrogen adducts by NMR spectroscopy.

In this work, we present a formula assignment procedure for the mass spectra of complex mixtures containing ^15^N and halogens. Our proposed method is robust and could be adapted to other isotopic labels. We then investigate the assigned formulas using network analysis and the PageRank algorithm.

Network analysis is a method of analyzing FT-ICR MS data by constructing a mathematical graph in which molecular formulas are nodes and the differences in atomic composition between them are edges. This type of graph is termed a “mass difference network”. Mass difference networks have been applied in previous studies to improve formula assignment,^32^ quantify chemical transformations,^33,34^ investigate reaction pathways,^35^ and predict metabolic networks.^36^

The use of mass difference networks offers the opportunity to apply network analysis algorithms developed in other disciplines to gain chemical insights. Two such algorithms explored in this work are PageRank and Reverse PageRank algorithms.

PageRank is an algorithm developed by Google in 1996 to rank search engine results.^37^ The algorithm simulates how Internet users navigate across a directed network of Web sites by clicking hyperlinks. PageRank produces a ranking of all of the Web sites in the network based on the probability that a given Internet user is viewing them. In the context of a mass difference network, where instead of Web sites there are molecular formulas and instead of hyperlinks there are (potential) chemical reactions, PageRank predicts the relative abundance of each formula in the network based on reaction pathways and does this independently of the peak intensity.

The Reverse PageRank algorithm^38^ is an attempt to tackle the inverse problem, i.e., the prediction of edge probabilities based on known PageRank scores. In the context of mass difference networks, this means calculating reaction probabilities from peak intensities associated with known molecular formulas.

In this work, we highlight how both algorithms can be applied to gain a unique insight into chloramination mass difference networks and suggest that these techniques could offer profound insights in other applications of FT-ICR MS.

Finally, we also present results using two additional labels: ^13^C and fluorine. The former consists of a ^13^C-labeled carboxylic group of a model compound used in this study, while the latter is its fluorinated analog. The use of ^13^C opens up avenues for NMR structure elucidation and F was chosen because of a rising prevalence of fluorine-containing persistent contaminants in drinking water,^39^ but also because we have performed ^19^F-centered NMR analysis of chloramination DBPs^29^ which gives us an opportunity to compare and validate the results obtained by two high-resolution techniques and the algorithms applied in this work.

## Experimental Section

### Materials

LC-MS grade water and methanol (Fisher Scientific) were used throughout the procedure. 4-Hydroxybenzoic acid (99%), 3-fluoro-4-hydroxybenzoic acid (95%), 4-hydroxybenzoic acid-α-^13^C (99%), ammonium-^15^N chloride (99%), and ^15^N-labeled amino acid mixture (5–100 mM in water) were purchased from Sigma-Aldrich. European Pharmacopoeia sodium hypochlorite solution standard (26 g/L) was obtained from Reagecon. Solid phase extraction (SPE) cartridges (Bond Elut PPL) were obtained from Agilent. Methanol-d_3_ (99.5% D) was purchased from Euroisotop.

### Sample Preparation

**Scheme 1:**
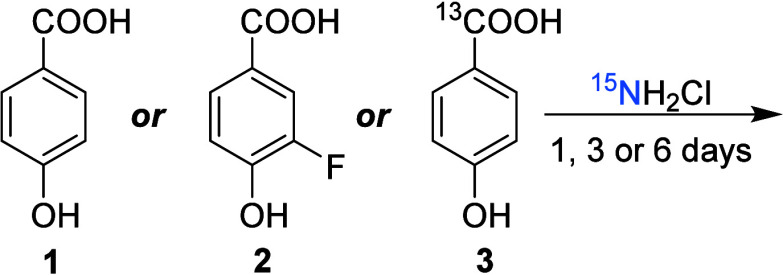
Starting Materials Used to Investigate Chloramine Disinfection Byproducts

The samples were prepared as described previously.^29^ 50 mg of 4-hydroxybenzoic acid (**1**) or 3-fluoro-4-hydroxybenzoic acid (**2**) (Scheme 1) were dissolved in 500 mL of water. Chloramine-^15^N solution was added, which had been prepared by dissolving 38 mg of ^15^NH_4_Cl in 2 mL of water and adding 1.6 mL of NaOCl solution (26 g/L) dropwise. This resulted in a carbon to active chlorine mass ratio of 3:5. The chosen concentration of model compounds (0.7 mM and 0.6 mM for **1** and **2**, respectively) and the disinfectant to compound ratio were in line with experimental conditions in previous MS and NMR studies of DBPs.^22,40,41^

The reaction mixture was split into 3 aliquots to be quenched with Na_2_S_2_O_3_ (100 mg), as commonly done in chloramine DBP studies,^22,40,41^ after 1, 3, or 6 days of reaction time at 20 °C in the dark. The quenched samples were acidified to pH 2 using HCl (1 M) and concentrated using SPE. PPL cartridges were chosen as they have been shown to have the highest extraction efficiency for DOM samples,^42^ and they have been used in extracting pollutants from water samples.^43,44^ Although HLB cartridges are often used in similar studies, we found that PPL worked better for our samples (Figure S1). The SPE cartridges were conditioned using methanol and acidified (pH 2) water; after sample loading, the column was once again washed with acidified water to prevent retention of inorganic compounds. The cartridge was dried and then eluted with MeOH (4 mL) to obtain the final product mixture. In order to account for compounds present in the starting material and SPE cartridges, control samples were prepared using the same chemicals; for these, chloramine was quenched before being added to the reaction vessel. The control sample was concentrated using the same SPE procedure.

### Mass Spectrometry

For FT-ICR MS analysis, a 0.2 mL aliquot of the MeOH SPE eluent was diluted 25-fold in MeOH/H_2_O (1:1). The remainder of the eluent was evaporated to dryness in a vacuum concentrator. The average yield was 12 mg of product mixture, resulting in an average concentration of 0.2 mg/mL for the MS samples.

Spectra were acquired using direct infusion electrospray ionization on a Bruker solariX 12 T FT-ICR mass spectrometer in negative mode. All spectra were obtained between 73.7 and 3000 *m*/*z*, with an ion accumulation time of 0.1 s; 100 scans of 2 MWord time domain size were summed for each analysis. The Q1 mass was 100 *m*/*z*, and the time-of-flight to the detector was set to 0.7 s. Three technical replicates were obtained for each sample; spectra were acquired in random order to minimize crossover effects.

Spectra were initially processed using Bruker DataAnalysis 4.0, data was calibrated externally to arginine clusters and internally to a list of compounds in Table S1. Peaks with signal-to-noise ratio >4 and an absolute intensity threshold of 10^6^ were picked and exported for further analysis.

### Formula Assignment

Molecular formulas were assigned using CoreMS.^45^ Since CoreMS searches for ^14^N first by default, the atomic masses of ^14^N and ^15^N were swapped. Additionally, the isotopic abundances were changed to match the enrichment of ^15^NH_4_Cl (98.2% ^15^N). Any reference to N in the following sections refers to ^15^N. This procedure could similarly be adapted to other complex mixtures using any isotopically enriched element by redefining the masses and abundances as for ^15^N in this case. An example CoreMS script is provided with the experimental data (see the Data Availability Statement).

Formulas were assigned using the following limits: C_29_H_72_O_18_N_10_Cl_8_ and C_29_H_72_O_18_N_10_F_3_Cl_8_ for reactions using **1** and **2**, respectively. Formulas of isotopologues containing lower-abundance isotopes (^13^C, ^14^N, ^18^O, and ^37^Cl) were also assigned if they were detected. The presence of S was ruled out by the lack of S isotopologue peaks in the spectra. The maximum allowed mass error was 1 ppm. The element limit restrictions were based on Rule #1 of the Seven Golden Rules;^46^ the F limit was a conservative 3 to allow for possible oligomerization of compound **2** and its derivatives, as it was found that a higher limit increased the chance of erroneous assignments. If multiple formulas were assigned to a single *m*/*z* value, the formula with the highest CoreMS confidence score was chosen. The confidence score accounts for mass error and isotopic pattern similarity (intensity and position of isotopologue peaks). Formulas found in control samples were excluded from analysis.

Data visualization was performed using PyKrev^47^ and UpSetPlot^48^ Python libraries, statistical parameters were calculated using SciPy,^49^ and network visualization was done using Cytoscape.^50^ A tutorial on how to perform PageRank and Reverse PageRank network analysis is available on the PyKrev GitHub page (https://github.com/Kzra/pykrev).

### NMR Spectroscopy

The sample for (IN)ADEQUATE NMR experiments was prepared following the procedure described above using 4-hydroxybenzoic acid-α-^13^C (**3**) as the starting material and a reaction time of 5 days. The resulting product mixture (28.4 mg) was dissolved in CD_3_OH (0.6 mL). NMR spectra were obtained on a Bruker 800 MHz spectrometer equipped with a TCI cryoprobe; the full details of the experimental parameters are given in the Supporting Information.

## Results and Discussion

### Formula Assignment

**Figure 1:**
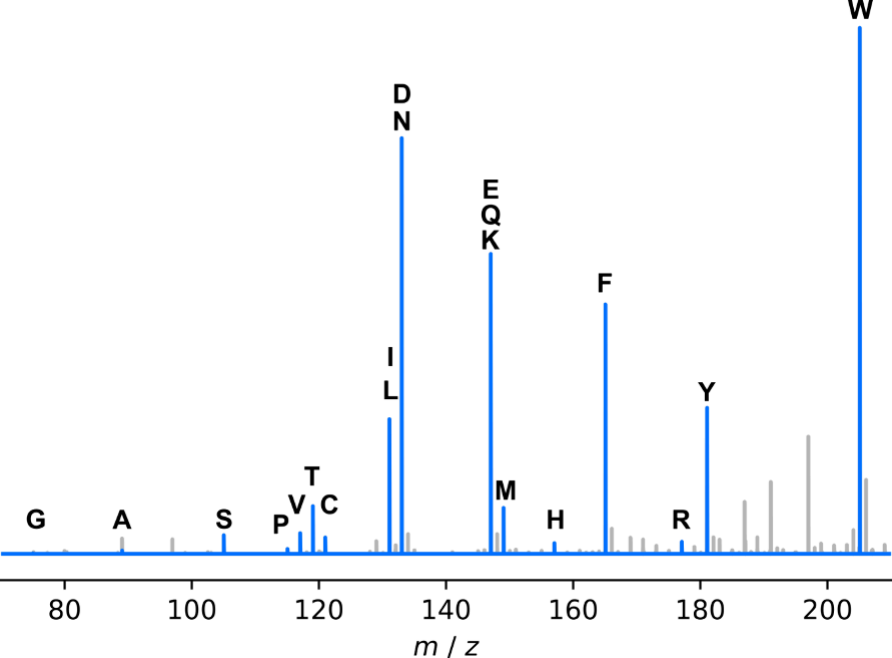
Negative mode ESI mass spectrum of 20 ^15^N-labeled amino acids. The amino acid peaks are highlighted in blue and labeled.

In order to ensure the modified formula assignment procedure of ^15^N-labeled compounds performed correctly, it was initially tested on a sample containing a mixture of 20 ^15^N-labeled amino acids. The assignment procedure was able to correctly assign the formula of all ^15^N-amino acids present in the sample (Figure 1). The full assignment list with ppm errors is given in Table S2. Additionally, the ^14^N isotopologues were assigned correctly, provided that they were above the detection limit. Therefore, we are confident that the CoreMS formula assignment procedure with the modifications to account for ^15^N labeling is working as expected, and thus, it was taken forward to assign formulas for a mixture of unknown DBPs. The performance of the procedure at higher *m*/*z* was validated by the correct assignment of amino acid adducts that were also present in the spectrum (Table S2).

### MS of Chloramination DBPs

**Figure 2:**
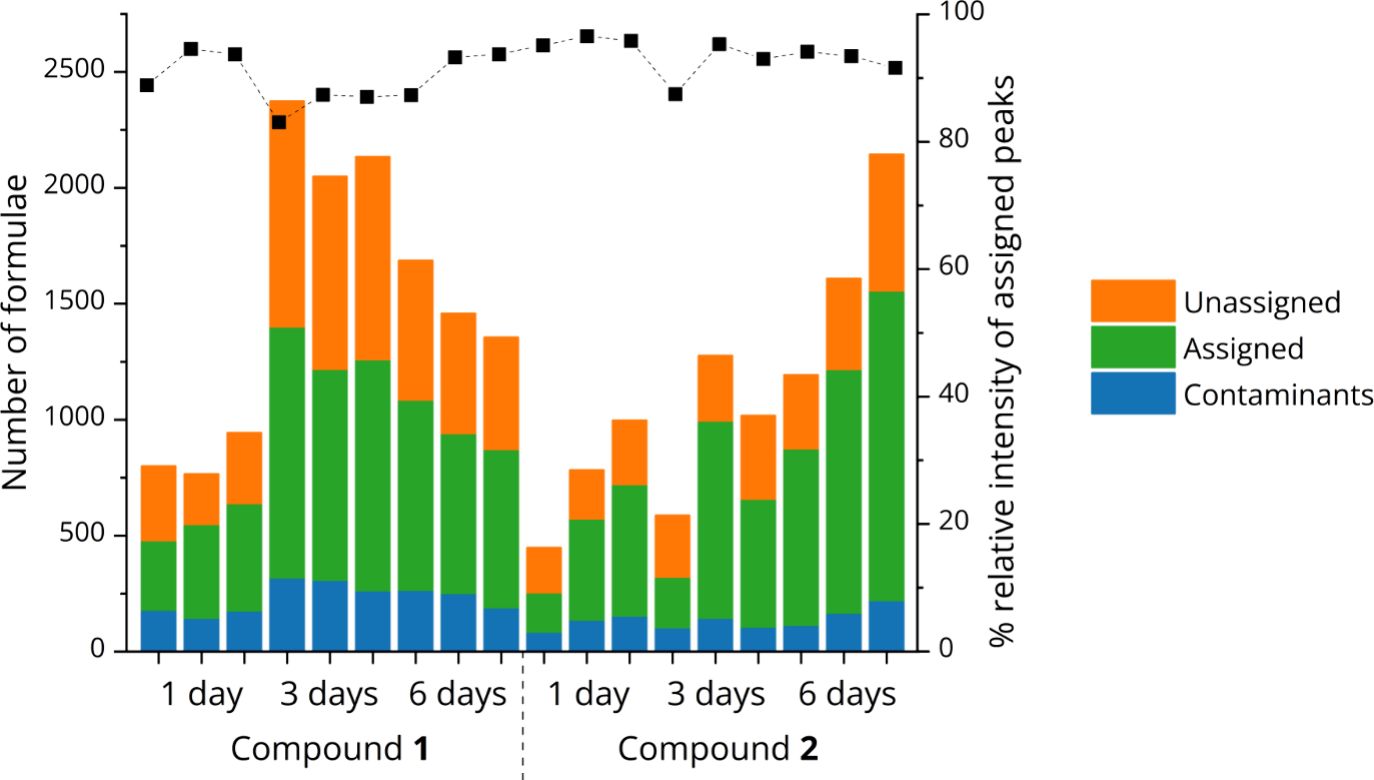
Number of peaks detected and formulas assigned for technical triplicates of three samples for compounds **1** and **2**. *Contaminants* refers to formulas present in control samples. The % relative cumulative intensity of the assigned peaks is shown by the scatter plot on the secondary axis.

A total of 6 samples of chloramination DBPs were investigated, prepared by reacting model compounds **1** and **2** with ^15^NH_2_Cl for 1, 3, or 6 days. The chosen time points were in line with other chloramine DBP investigations^22,41^ and cover a range of drinking water residence times in distribution systems.^51^ Three technical replicates of each sample were obtained, resulting in 18 spectra being analyzed. Between 449 and 2375 peaks with a signal-to-noise ratio >4 were detected in the samples. The number of peaks with assigned formula is shown in Figure 2. The assigned peak *m*/*z* ranged between approximately 100 and 1100. The unassigned peaks mostly correspond to *m*/*z* values with unusual mass defects. These are likely due to inorganic contaminants, the majority of which were removed during SPE (Figure S2), and double or triple charged compounds. Additionally, there are some unassigned bromine-containing compounds due to the presence of bromide impurities in the reagents used. Nevertheless, the unassigned peaks make up the minority of the overall ion count, as determined by the relative intensity of the assigned peaks—this was on average 89%, although this does not necessarily mean that 89% of the contents of the sample were assigned formulas since MS intensity depends on ionization efficiency.

**Figure 3:**
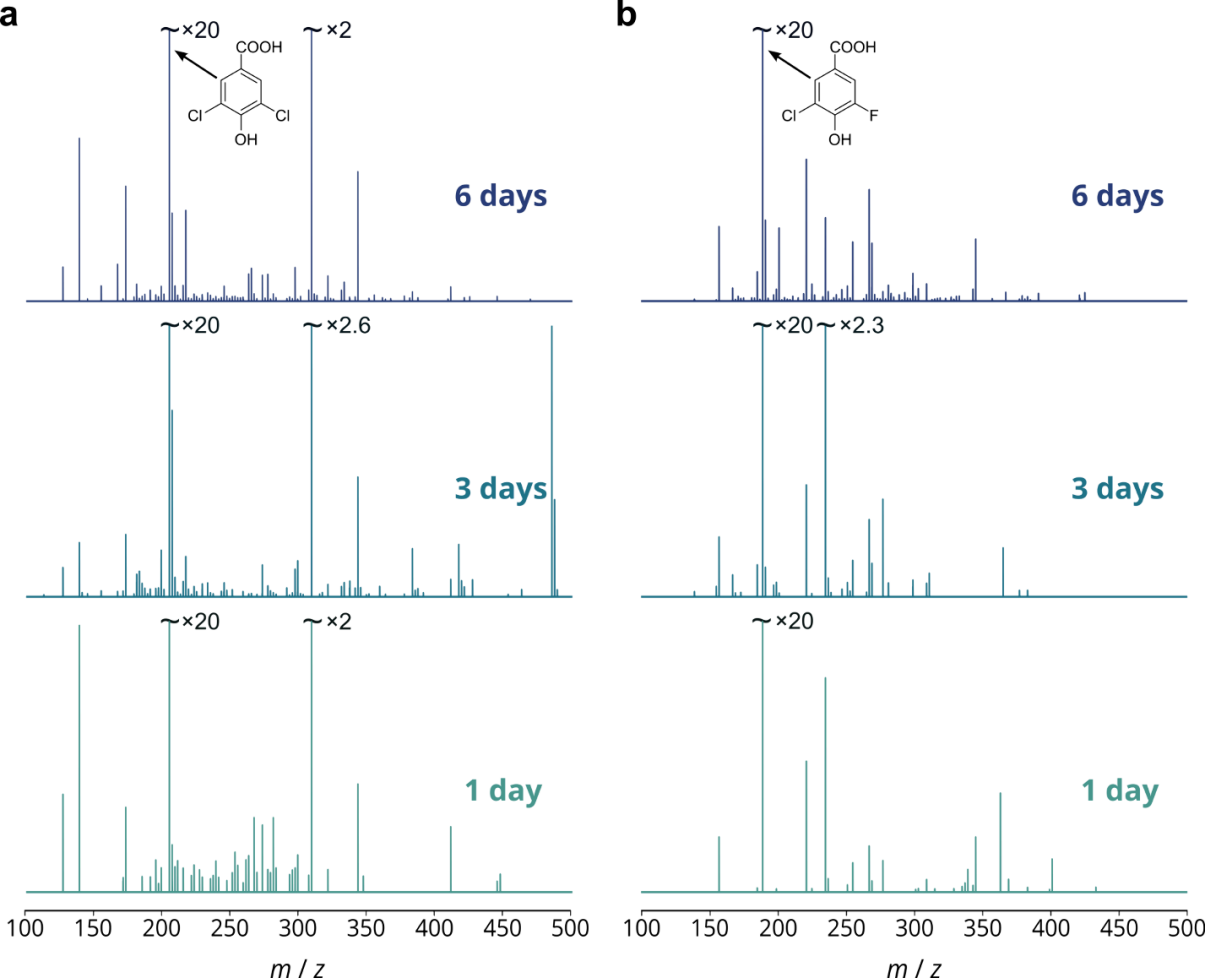
Reconstructed mass spectra of chloramine DBPs of (a) **1** and (b) **2** after 1, 3, and 6 days of reaction time. Only formulas present in all 3 technical replicates are plotted, and their intensities are averaged (mean). Spectra are normalized to the highest intensity peak in each spectrum corresponding to the shown structural formulas.

The difference in the mean number of formulas assigned was tested for significance in terms of starting material and reaction time using two-way ANOVA. The number of formulas did not differ significantly between reactions of **1** and **2** (*p* = 0.55); however, the number of formulas differed significantly between different time points (*p* = 0.01). Figure 2 also indicates that there is a variation between technical replicates of the same sample. Analysis of their molecular formulas showed that a large number is unique, and only 11% on average are identical between all 3 technical replicates of the same sample. This could be explained by ion suppression, crossover effects, or the DBPs reacting among themselves and/or decomposing after the chloramine is quenched. Therefore, in order to increase confidence of the analysis, only the formulas present in all 3 replicates were taken forward to further analysis. The reconstructed mass spectra corresponding to these molecular formulas are shown in Figure 3.

**Figure 4:**
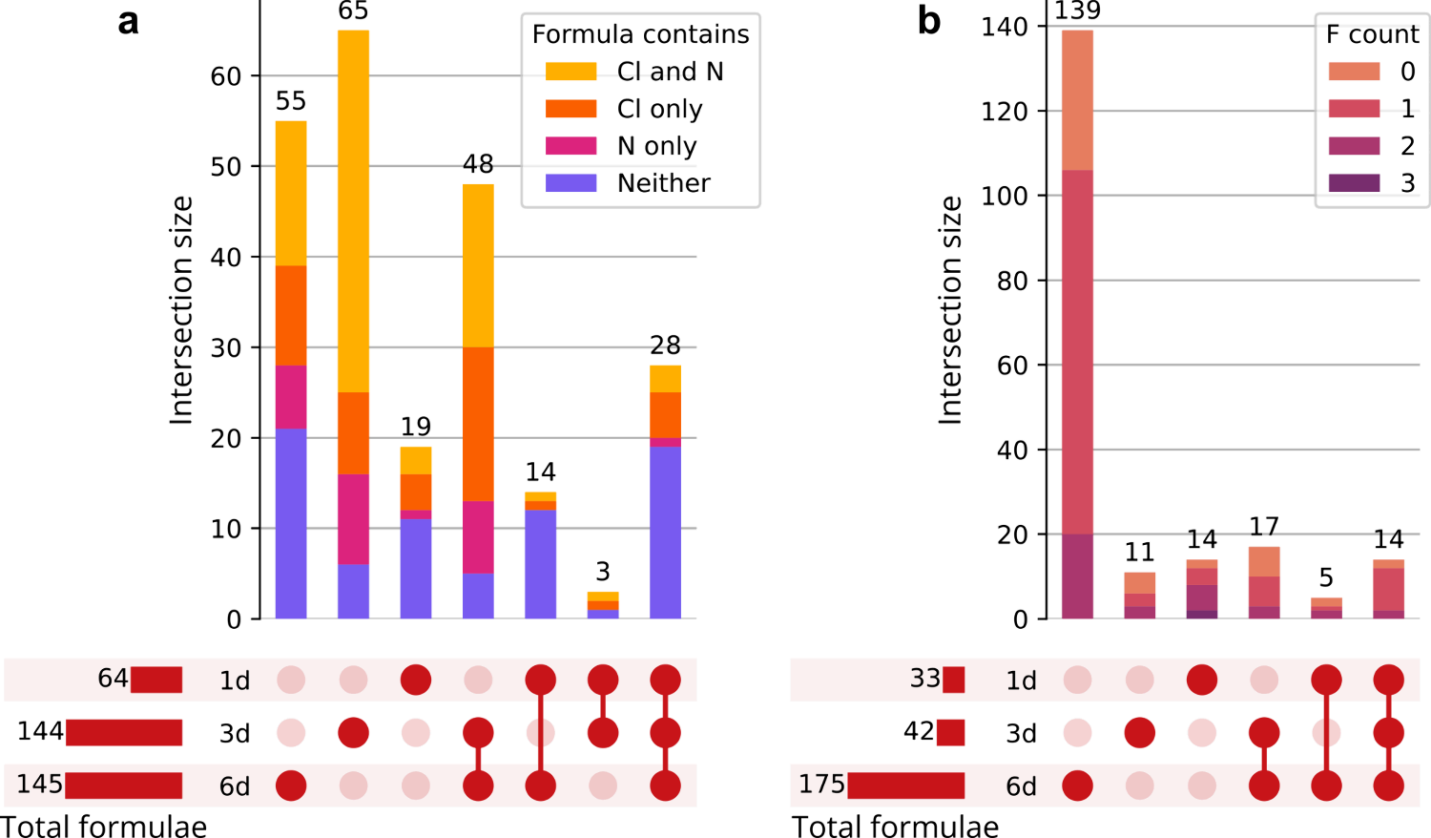
UpSet plots of chloramination DBPs for days 1, 3, and 6 samples of (a) **1**, colored by N and/or Cl content and (b) **2**, colored by number of F atoms.

The number and relationship between the formulas present at different time points are shown in the UpSet plots of Figure 4. There is an increase in the number of compounds present as time progresses. Interestingly, the number of compounds in the reaction of **1** increases quicker than that of **2**; this could be due to the slightly deactivating effect of the fluorine on the aromatic ring. A vast majority (90% for **1**, all for **2**) of compounds present in both day 1 and day 3 samples are also present in the day 6 sample; these are likely the major products that form early and stay abundant during the progress of the reaction. Despite the potential for the addition of chlorine and nitrogen, some of the detected compounds do not contain either of those elements (Figures 4a and S3). This suggests that chloramine catalyzes or generates an environment favoring other chemical transformations that do not involve the addition of N or Cl, such as phenol oxidation to quinones. Although most DBPs of **2** contained one fluorine atom (Figure 4b), some formulas were identified with 0 or 2–3 F atoms. This indicates that chloramine can cause the release of F^–^, which can then rapidly react with other constituents of the reaction mixture, producing difluoro compounds. F^–^ has been long known as the best leaving group in nucleophilic aromatic substitution reactions,^52^ a reaction type which has been identified in chloramination model systems.^23^ Compounds containing more than one fluorine atom are also formed through oligomerization reactions of the starting material, such as esterification and ether formation. The presence of oligomerization is evident from the mean *m*/*z* of the mono- and difluorinated compounds; these were 258 and 317, respectively, in the 6-day sample, indicating that the difluoro compounds are significantly larger (*t*-test: *p* < 0.001).

### Statistical Analysis

In the chloramination of **1**, the number of N and Cl atoms differed significantly between different time points (one-way ANOVA: *p* < 0.001 for both). Tukey posthoc testing indicated that the difference in Cl was significant between all pairs of samples (*p* = 0, 0.002, 0.013), meaning that Cl incorporation from chloramine plays a significant part in the generation of DBPs. The N count in the three-day sample differed significantly from the other two samples (*p* < 0.001 for both). This shows that the nitrogen number increases rapidly at the start of the reaction but N-containing groups are lost as the reaction continues. For reaction of **2**, the N and Cl number in the detected DBPs did not differ significantly between different time points (Cl – *p* = 0.31, N – *p* = 0.49), while the F number differed significantly (*p* = 0.02). Tukey posthoc testing indicated that the significant differences were between the one-day and three-day (*p* = 0.04), and one-day and six-day (*p* = 0.02) samples. This indicates that fluorine could play a significant part in the starting phase of the reaction; however, its importance decreases as the reaction progresses.

The mean *m*/*z* differs significantly between days for reaction of **1** (one-way ANOVA: *p* = 0.007) and **2** (*p* < 0.001). In the case of **1**, the one- and three-day sample differ significantly from each other’s points (Tukey: *p* = 0.01); however, the mean *m*/*z* of the six-day sample does not differ from the other two time points. This indicates there is an initial peak of reactions significantly increasing the mass (such as esterification or addition of Cl); however, this is then countered by reactions that degrade the DBPs into smaller fragments. For reaction of **2**, the mean *m*/*z* of the one-day sample differs significantly from the three- and six-day samples (Tukey: *p* < 0.001 and *p* = 0.02, respectively). The results may be affected by the low mass limit of FT-ICR MS; it is possible that there are low molecular mass compounds not being detected by the technique, including regulated compounds such as haloacetic acids.

**Figure 5:**
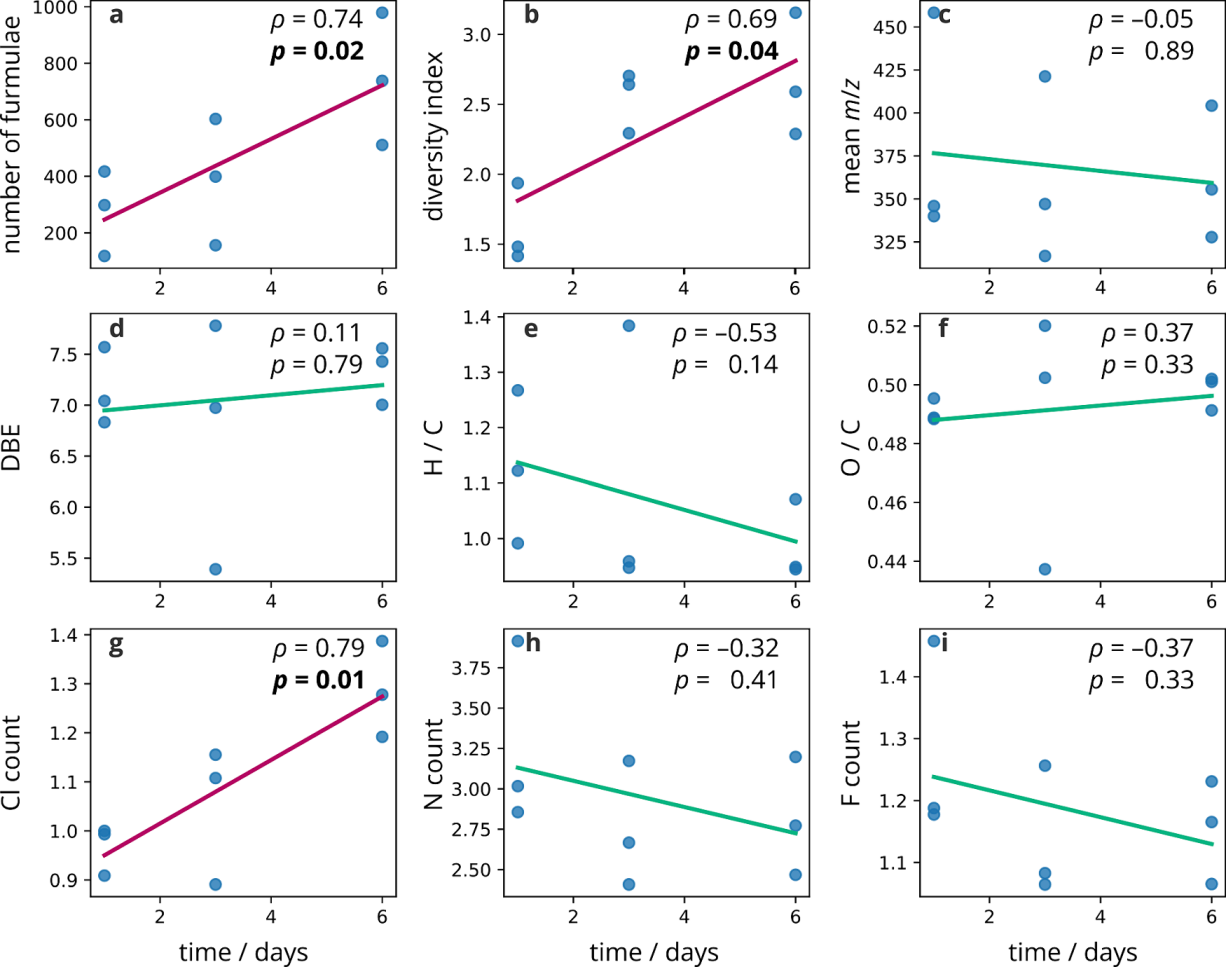
Correlation for chloramination of **2** of (a) average number of formulas, (b) Shannon diversity index; (c) mean *m*/*z*, (d) double bond equivalent, (e) hydrogen-to-carbon ratio, (f) oxygen-to-carbon ratio, and (g–i) count of Cl, N, and F against time. Correlations were determined using Spearman’s rho (ρ), and statistically significant correlations are highlighted in red. Each point represents a technical replicate.

The correlation of various formula metrics—such as number of formulas, mean *m*/*z*, aromaticity index, double bond equivalent, elemental ratios, and element counts—with time was investigated. For this analysis, the three technical replicates were treated separately, and the average values of the metrics were calculated for each replicate (Tables S3 and S4). Thus, this analysis includes formulas that are not present in all 3 technical replicates. Unweighted averages were used in order to obtain a picture of all the formulas present in the sample without being overshadowed by one or two major products. A comparison between using weighted and unweighted Cl means is presented in Figure S5. The correlation was determined using Spearman’s ρ coefficient. In the reaction of **2** (Figure 5), the average number of formulas and the average chlorine count in the compounds significantly correlated with time (*p* = 0.02, 0.01, respectively), indicating that the complexity of the reaction and incorporation of Cl increases as the reaction progresses. The correlation plots of **1** are shown in Figure S4.

### Chemical Diversity and Network Analysis

The chemical diversity of the samples was investigated using the Shannon diversity index.^53^ This method has been applied to FT-ICR MS studies of DOM previously.^54^ The higher the index, the higher the diversity of the sample. The lowest possible value is 0, which is when the abundance of all but one species is zero. When applied to MS, the peak intensity is used to indicate species abundance. We note that this assumes that mass spectra are quantitative and that peak intensity is proportional to the concentration. This is not the case, as the peak intensity is affected by ionization efficiency.^55^ Additionally, for samples with compounds of unevenly distributed concentration, ion suppression could take place since ionization is a competitive process, meaning that compounds of lower concentration are less likely to be ionized. Nevertheless, the Shannon index is a useful metric to qualify the diversity of ion abundance in mass spectra. The Shannon diversity index showed a significant correlation with time for chloramination of both compounds **1** and **2** (*p* = 0.004 and 0.04, respectively); however, the sign of these correlations differs. This is due to the Shannon index accounting for not only the number of peaks but also an increasing spread of peak intensities rather than the spectrum being dominated by one major peak. Therefore, the Shannon diversity index decreases with time for **1**, despite the number of formulas increasing, since the spectra become dominated by the major products. To the contrary, the diversity index increases with time for the reaction of **2** (Figure 5b), indicating that the abundance of the nonmajor products is growing with time. The larger value of the Shannon diversity index for compound **1** indicates that the chemodiversity of its products is significantly higher than for **2** (paired *t*-test, *p* = 0.05), although the difference in number of formulas was not significant. This is also apparent from the mass spectra shown in Figure 2: the major products in the reaction of **2** are more intense than the remaining products, particularly for days 1 and 3. Caution needs to be taken when comparing diversity trends, as this could directly translate to compound concentrations in the samples; however, it could be caused by ionization differences in the two samples. It is possible that the major products of **2** are more likely to cause ion suppression, and hence appear more abundant than they are in the sample. The Shannon index can also be calculated for the ^19^F NMR spectra of **2** (Figure S6). As with the MS data, both the number of peaks and the Shannon index increase with time. Note that the absolute value of the index is lower than that of the mass spectra as there are much fewer peaks in the NMR spectra. Overall, the conclusions drawn from the Shannon index show that it is a useful metric to quantify spectral diversity, as it might be difficult to visually discern how peak numbers and intensity distributions differ in complex spectra.

**Figure 6:**
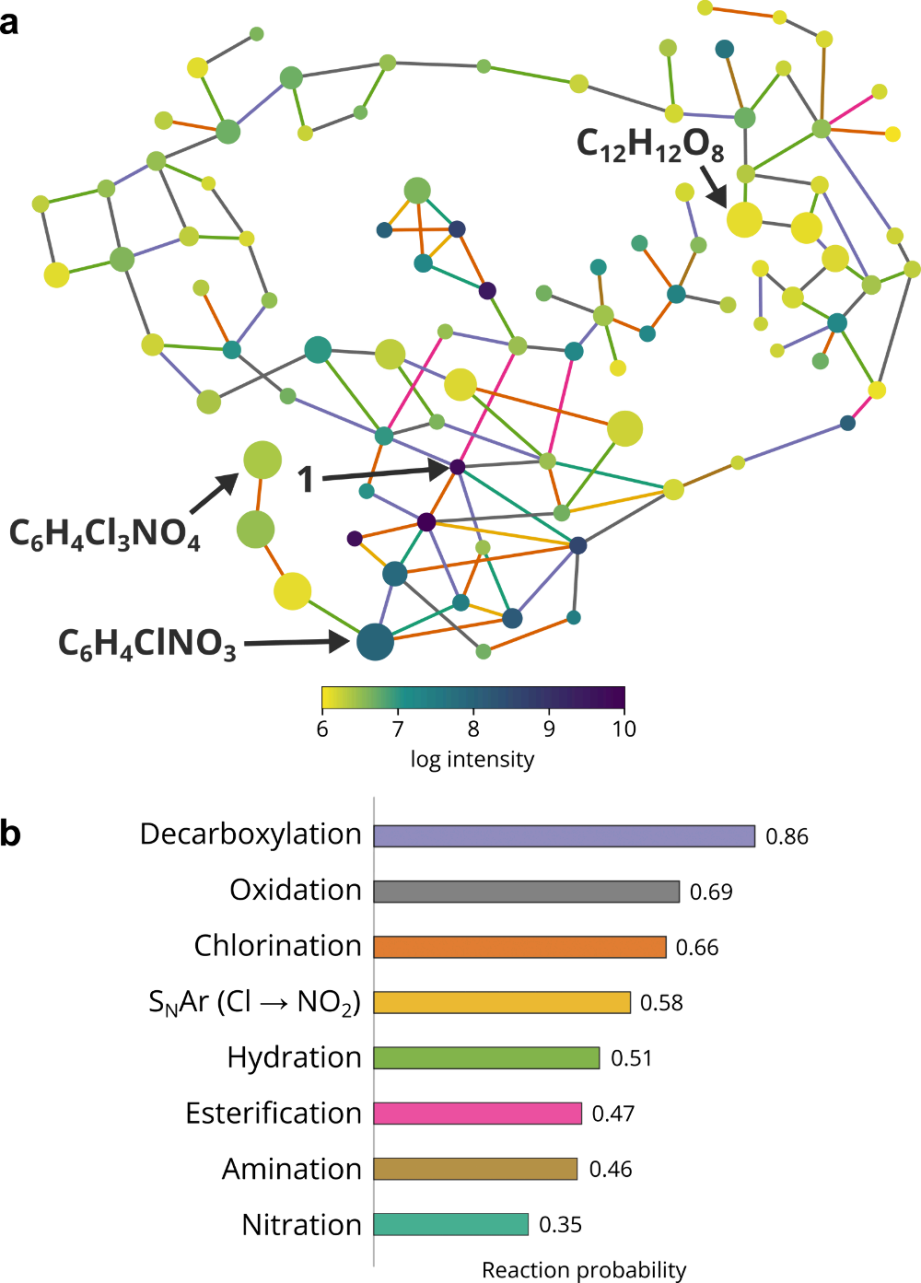
(a) Reaction network of chloramination DBPs of **1** after 6 days of reaction time. The nodes are scaled to their PageRank score and colored according to their MS intensity (log scale). The nodes are linked by edges, which represent different reaction types. The starting material (**1**) and formulas with highest PageRank score are labeled. (b) The Reverse PageRank reaction probabilities are shown for each reaction type. S_N_Ar–nucleophilic aromatic substitution.

The different types of reactions taking place during chloramination were also analyzed by using a network analysis. The PageRank algorithm, initially developed by Google to rank search engine results,^37^ was used to identify formulas of the reaction network with the highest probability at convergence of the algorithm. Here, the term convergence is used in a statistical rather than a chemical context, that is, that these formulas are likely to be formed at higher abundance than others and may suggest they are more stable; however, it does not imply that the reaction pathway converges to just these formulas. PageRank probabilities are based solely on the reaction network topology and are independent of peak intensities. The reaction network consists of nodes and edges, which represent formulas and reaction types, respectively, as shown in Figure 6. The reaction types are defined by the user by specifying the elemental change and hence the change in mass. The reactions considered in this network were decarboxylation (CO_2_ → H), hydration (+H_2_O), oxidation (+O), chlorination (H → Cl), nitration (H → NO_2_), amination (+NH), nucleophilic aromatic substitution (Cl → NO_2_), and esterification with 1 equiv of the starting material. For **2**, defluorination (F → H) was also considered. These transformations were chosen as they have been identified in the literature or were evident from preliminary analysis of mass spectra. The chemical diversity analysis was performed on the formulas present only in all three technical replicates of the six-day sample.

For the reaction of **1**, the formulas with the highest probability at convergence were identified as C_12_H_12_O_8_ and C_6_H_4_Cl_3_NO_4_. The first one is likely a heavily oxidized ester derivative of the starting material, not containing Cl or N atoms. The second is likely a heavily chlorinated derivative in which the carboxylic acid group was replaced by a nitro group. This analysis shows the usefulness of performing network analysis—although we only have the chemical formulas of compounds the reaction is likely to converge to, by following the chemical transformations in the network map we are able to make sensible predictions about their structures. To confirm that these compounds are present in the sample, validation using MS/MS could be insightful. Due to the crowded nature of the complex mixture spectra, there was insufficient separation of peaks corresponding to the convergence molecular formulas, and quadrupole isolation followed by CID was not possible. We were able to isolate C_6_H_4_ClNO_3_, the fifth highest ranking formula, in the ICR cell using sweep and shot pulses (Figure S7), however our efforts to fragment it using EID were unsuccessful.

For the reaction of **2** (network presented in Figure S8), the two highest ranking formulas were C_6_H_4_Cl_2_FNO_4_ and C_12_H_6_O_2_F_2_Cl_2_—nitrated and chlorinated derivatives of the starting material and its dimer, respectively. The network analysis of **2** reveals that there are instances of formulas differing by only one F atom. This corroborates the theory that F^–^ is released during the reaction.

The Reverse PageRank algorithm^38^ can provide additional useful information about the reaction network. In this case, the relative abundances of the nodes (peak intensities) are used in order to determine which type of edges (reaction types) is the most probable. Performing this analysis, the highest reaction probability for **1** is decarboxylation (*P* = 0.86), oxidation (*P* = 0.69), and chlorination (*P* = 0.66). For compound **2**, the same reaction types ranked highly and nucleophilic aromatic substation of Cl by NO_2_ (via a short-lived undetected amine intermediate) was calculated to have the highest probability (*P* = 0.81). Decarboxylation and chlorination reactions have been identified in a previous study,^29^ and chloramine, like all water disinfectants, is an oxidant,^30^ explaining the oxidation products. The findings also agree with the experimental observation that nitration happens through substitution and not a direct addition mechanism. A possible limitation of the Reverse PageRank approach is that once again, the peak intensities are assumed to be directly quantitative. Nevertheless, since the most probable reaction types identified by this analysis agree with literature data, Reverse PageRank could be a useful technique to study the dynamics of unknown complex mixtures.

### Structure Elucidation Using NMR

Although the FT-ICR MS analysis provided useful insights into the reactions that generated a complex mixture of DBPs, it did not reveal information about their structures, particularly without the use of MS/MS. To investigate the structure of a subset of DBPs, we turned to NMR spectroscopy, focusing on compounds that retained the carboxylic group. Following the strategy of utilizing ^13^C labeled atoms for structure determination of compounds contained in the complex mixture,^56,57^ we have repeated the reaction starting with compound **3** containing 100% ^13^C-labeled carboxylic acid group. The produced labeled DBPs stand out from the rest of the mixture and make it possible to utilize NMR experiments that trace the carbon skeleton of molecules. These experiments are not used routinely in the structure elucidation of organic molecules with natural abundance of ^13^C due to their relatively low sensitivity.

**Figure 7:**
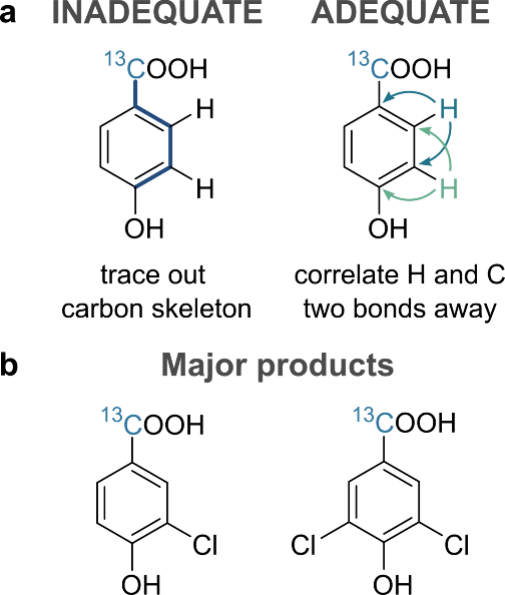
(a) Structural information provided by INADEQUATE and ADEQUATE NMR experiments. (b) The structures of the major products of chloramination of **3** as identified by NMR spectroscopy.

The ^13^C 1D NMR spectra (Figure S9) revealed the presence of two major products in the carboxylic acid region present at a high concentration. The structures of these two major products were investigated. We used INADEQUATE^58^ and a sensitivity enhanced variant^59^ of ADEQUATE.^60^ The INADEQUATE experiment correlated the chemical shift of carbons separated by 1- to 4-bonds, while ADEQUATE optimized for ^1^*J*_CC_ correlations complemented the data by tracing out 2-bond H–C correlations. Considering the effect of substituents on the chemical shifts of the starting material, the obtained correlated proton and carbon chemical shifts were interpreted to identify the structure of the two major products of chloramination of **1**, which correspond to the two highest intensity peaks in the mass spectra in Figure 2a. These were the mono- and dichlorinated derivatives of the starting material (Figure 7). The full INADEQUATE and ADEQUATE spectra are presented in Figure S10, and the chemical shift assignments are shown in Figure S11. We were unable to identify any of the highly ranked compounds from the PageRank algorithm as their production involves the loss of the ^13^C label via decarboxylation. The results of NMR analysis confirmed that the reaction probabilities predicted by Reverse PageRank were accurate—chlorination was one of the most probable reaction types. Although nitration and decarboxylation were given a higher probability, these reactions involve the loss of the ^13^COOH group, and hence, their products could not have been detected by (IN)ADEQUATE experiments. Therefore, it was only possible to analyze products that retained the ^13^COOH group; however, the use of a label within the aromatic skeleton of the molecule could be the way to go forward for the validation of an extended set of products, and our experiments indicate that this approach would be fruitful. The presence of nitration is nevertheless confirmed by the ^1^H, ^15^N HMBC spectrum (Figure S12), which contains multiple peaks in the nitro group region at 340–390 ppm.^61^ Finally, a high Reverse PageRank probability does not necessarily mean that the concentration of a single product of that reaction will be the highest; rather it is a reflection of the total concentration of all products that have undergone that reaction. Overall, the NMR data corroborate the Reverse PageRank findings and show that the information obtained from the algorithm predictions could be useful in deciding what type of NMR experiments should be acquired or developed for structure elucidation of complex mixtures.

### PageRank Limitations

The PageRank technique has limitations that need to be articulated, in particular for our system, where the high PageRank formulas have not been confirmed analytically. In general, it is impossible to consider every scenario; therefore, the algorithm is constrained to sensible reactions that we know are likely. Moreover, it is possible that the proposed highly abundant products react further via a pathway that we have not considered in the network. The more well-understood the system, the more accurate the network topology can be considered. Highly dynamic complex mixtures will always pose a challenge. The work presented here is valuable nevertheless, as it makes testable predictions about the complex mixture, and it opens doors for future work in developing the algorithm by using a well-known product to optimize it further. Alternatively, the algorithm could be improved iteratively through tweaking the network topology after confirming/denying the existence of certain validated compounds.

## Conclusions

We have produced a formula assignment strategy for the mass spectra of complex mixtures containing ^15^N and halogens. This strategy allowed the products of chloramination of 4-hydroxybenzoic acid and 3-fluoro-4-hydroxybenzoic acid to be followed over the course of 6 days. Statistical analysis showed that the complexity of the DBPs increased with time but it was not significantly affected by starting material. The reaction products were also investigated in a reaction network, and informatics-inspired algorithms were used to gain information about the transformations taking place without the need to elucidate the structures of the compounds. The PageRank algorithm showed that the reaction is likely to converge to highly chlorinated, oxidized, and nitrated products; and similarly, the Reverse PageRank algorithm revealed that chlorination and decarboxylation are the most likely reaction types in chloramine DBP formation. Using NMR, the structure of the major DBPs was elucidated to be mono- and dichlorinated derivatives of the starting material, agreeing with the network analysis. Overall, these methods show promise in the field of complex mixtures and could be transferred to any isotopically labeled mixture even without prior knowledge of the reactions taking place.

## Data Availability

The data underlying this study, including NMR and mass spectra, MS formula lists, and Python scripts used for formula assignment and data analysis, are openly available at https://doi.org/10.7488/ds/7733.

## Supporting Information Available

Additional experimental details, reaction networks and NMR spectra (PDF)
