# Supplementary material for: MS and NMR Analysis of Isotopically Labeled Chloramination Disinfection Byproducts: Hyperlinks and Chemical Reactions
Source: Anal Chem. 2024 May 9;96(21):8263–72. doi: 10.1021/acs.analchem.3c03888 (PMC11140672; doi:10.1021/acs.analchem.3c03888)
Supplement: Supplementary file 1 — ac3c03888_si_001.pdf [file ac3c03888_si_001.pdf]

Supporting Information

MS and NMR analysis of isotopically-labelled chloramination disinfection by-products: hyperlinks and chemical reactions

Justinas Sakas, Ezra Kitson, Nicholle G. A. Bell, Dušan Uhrín

EaStCHEM School of Chemistry, University of Edinburgh, David Brewster Rd, Edinburgh, EH9 3FJ.

Contents

1. NMR experimental parameters S2
2. MS calibration lists S2
3. ^{15}N-labelled amino acid assignment S3
4. Summary of formula metrics S4
5. Comparison of PPL and HLB SPE cartridges S5
6. Comparison of mass spectra of **2** throughout sample workup S5
7. UpSet plot of **2** S6
8. Correlation plots of **1** S6
9. Comparison of weighted and unweighted means S7
10. Shannon index of ^{19}F NMR spectra of **2** S7
11. Isolation of high-PageRank product of **1** S8
12. Reaction network of **2** S8
13. ^{13}C NMR analysis of **3** S9
14. INADEQUATE and ADEQUATE NMR spectra of **3** S10
15. ^{1}H, ^{15}N HMBC spectrum of **3** S11

References S11

1. NMR experimental parameters

NMR spectra were recorded on a Bruker Avance Neo 800 MHz spectrometer equipped with a TCI triple-resonance, *z*-gradient cryoprobe.

1D ^{13}C ^{1}H-decoupled spectra were obtained using the Bruker *zgpg* pulse sequence. The spectrometer offset was set to 100 ppm. 65,536 time domain points were acquired with a spectral width of 202 ppm, resulting in an acquisition time of 0.81 s. The relaxation delay was 3 s. A total of 2048 scans was accumulated, resulting in an experiment time of 2 hours 14 minutes. The processed spectrum size was zero filled to 131,072 points and apodized using an exponential window function with line broadening of 2 Hz.

2D INADEQUATE spectra were recorded using the Bruker *inadgpqfsp* pulse sequence. The J_{CC} evolution delay was optimised for long-range coupling constants of 5 Hz. 32,768 and 768 time domain points were acquired in F_2 and F_1, respectively. 4 scans were acquired per increment. The spectral width was 100 ppm in both dimensions, corresponding to acquisition times of 819 ms and 38 ms in F_2 and F_1, respectively. The offset was set to 145 ppm in both dimensions. The total experiment time was 2 hours 33 minutes. The processed spectrum size was 65,536×2048 points with zero filling in both dimensions. Both dimensions were apodised using a cosine square window function.

2D ADEQUATE spectra were recorded using the pulse sequence published previously.[1] The J_{CH} evolution delay was optimised for 160 Hz, the J_{CC} evolution delay was optimised for one-bond coupling constants of 60 Hz. 2048 and 40 time domain points were acquired in F_2 and F_1, respectively. 320 scans were acquired per increment. The spectral width was 7.8 ppm and 70 ppm in F_2 and F_1, respectively, corresponding to acquisition times of 164 ms and 1.4 ms in F_2 and F_1, respectively. The offset was set to 6.5 ppm and 140 ppm in F_2 and F_1, respectively. The total experiment time was 6 hours 18 minutes. The processed spectrum size was 4096×512 points with zero filling in F_2 and forward linear prediction in F_1. Both dimensions were apodised using a cosine square window function.

2. MS calibration lists

Table S1. Calibration lists used for internal calibration of samples of reactions **1** and **2.**[a]

**1**		**2**	
Formula	m/z	Formula	m/z
$C_7H_5O_3^-$	137.02442	$C_7H_4FO_3^-$	155.01500
$C_7H_4ClO_3^-$	170.98545	$C_7H_3ClFO_3^-$	188.97602
$C_7H_6ClO_3^-$	173.00110	$C_7H_5ClFO_3^-$	190.99167
$C_7H_3Cl_2O_3^-$	204.94647	$C_7H_3BrFO_3^-$	232.92551
$C_{14}H_{11}O_6^-$	275.05611	$C_{16}H_{31}O_2^-$	255.23295
$C_{14}H_{10}ClO_6^-$	309.01714	$C_{14}H_9F_2O_6^-$	311.03727
$C_{19}H_{37}O_4^-$	329.26973	$C_{19}H_{37}O_4^-$	329.26973
$C_{21}H_{41}O_4^-$	357.30103	$C_{19}H_{38}ClO_4^-$	365.08494

[a] The spectra were externally calibrated to arginine clusters; the internal calibrations lists were only used to fine tune the calibration.

3. ^{15}N-labelled amino acid assignment

Table S2. List of amino acid formula assignment with mass error in ppm.[a]

Amino acid		Formula	Exp. m/z	Calc. m/z	Error / ppm	Intensity / 10^6
Glycine	G	$C_2H_5NO_2$	75.02180	75.02179	0.176	0.5
Alanine	A	$C_3H_7NO_2$	89.03745	89.03744	0.148	4.3
Serine	S	$C_3H_7NO_3$	105.03236	105.0324	0.081	23.9
Proline	P	$C_5H_9NO_2$	115.05310	115.0531	0.114	6.4
Valine	V	$C_5H_{11}NO_2$	117.06875	117.0687	0.111	26.4
Threonine	T	$C_4H_9NO_3$	119.04801	119.0480	0.071	60.7
Cysteine	C	$C_3H_7NO_2S$	121.00952	121.0095	0.099	20.9
Isoleucine / leucine	I/L	$C_6H_{13}NO_2$	131.08439	131.0844	0.023	171.0
Aspartic acid	D	$C_4H_7NO_4$	133.02727	133.0273	0.030	527.4
Asparagine	N	$C_4H_8N_2O_3$	133.04029	133.0403	0.035	73.5
Glutamic acid	E	$C_5H_9NO_4$	147.04292	147.0429	0.026	380.7
Glutamine	Q	$C_5H_{10}N_2O_3$	147.05594	147.0559	0.031	108.3
Lysine	K	$C_6H_{14}N_2O_2$	147.09233	147.0923	0.061	3.2
Methionine	M	$C_5H_{11}NO_2S$	149.04082	149.0408	0.080	58.5
Histidine	H	$C_6H_9N_3O_2$	157.05331	157.0533	0.034	13.7
Phenylalanine	F	$C_9H_{11}NO_2$	165.06875	165.0687	0.079	316.4
Arginine	R	$C_6H_{14}N_4O_2$	177.09255	177.0925	0.064	15.4
Tyrosine	Y	$C_9H_{11}NO_3$	181.06366	181.0637	0.047	185.4
Tryptophan	W	$C_{11}H_{12}N_2O_2$	205.07667	205.0767	−0.004	667.4
Cystine		$C_6H_{12}N_2O_4S_2$	241.01066	241.0106	0.073	336.6
	D_2	$C_8H_{14}N_2O_8$	267.06182	267.0618	0.042	183.0
	DE	$C_9H_{16}N_2O_8$	281.07745	281.0775	−0.031	174.9
	E_2	$C_{10}H_{18}N_2O_8$	295.09311	295.0931	0.004	78.6
	DR	$C_{10}H_{21}N_5O_6$	311.12708	311.1271	−0.004	96.0
Adducts	ER	$C_{11}H_{23}N_5O_6$	325.14273	325.1427	−0.004	77.6
	LW	$C_{17}H_{25}N_3O_4$	337.16833	337.1683	−0.013	73.8
	FW	$C_{20}H_{23}N_3O_4$	371.15271	371.1527	0.069	54.0
	YW	$C_{20}H_{23}N_3O_4$	387.14764	387.1476	0.106	52.7
	W_2	$C_{22}H_{24}N_4O_4$	411.16061	411.1606	−0.020	119.0

[a] All acids were fully ^{15}N-labelled, hence N in the table refers to ^{15}N (m = 15.00011).

4. Summary of formula metrics

Table S3. Summary of formula metrics for the samples analysed.

Sample[a]	Number of formulae	Shannon diversity index	*m*/*z*[b]	DBE[b]	H/C[b]	O/C[b]	Cl[b]	N[b]	F[b]
**1**-1-1	217	3.2	419	8.0	1.27	0.49	0.9	3.0	
**1**-1-2	366	3.9	321	8.1	0.98	0.68	0.3	1.1	
**1**-1-3	399	3.8	325	8.2	1.01	0.62	0.4	1.7	
**1**-3-1	738	3.8	471	7.9	1.32	0.48	1.4	3.7	
**1**-3-2	647	3.0	399	9.4	0.96	0.56	1.5	3.0	
**1**-3-3	792	3.1	343	9.2	0.91	0.54	1.0	2.8	
**1**-6-1	560	3.0	446	8.4	1.21	0.49	1.3	3.2	
**1**-6-2	477	2.9	411	8.8	1.08	0.55	1.2	3.1	
**1**-6-3	529	2.8	331	9.0	0.89	0.56	0.8	2.8	
**2**-1-1	118	1.4	458	7.0	1.27	0.50	1.0	3.9	1.5
**2**-1-2	298	1.5	340	7.6	0.99	0.49	1.0	2.9	1.2
**2**-1-3	417	1.9	346	6.8	1.12	0.49	0.9	3.0	1.2
**2**-3-1	156	2.7	421	5.4	1.38	0.44	0.9	3.2	1.3
**2**-3-2	603	2.3	347	7.8	0.95	0.52	1.1	2.7	1.1
**2**-3-3	399	2.6	317	7.0	0.96	0.50	1.2	2.4	1.1
**2**-6-1	511	2.3	404	7.4	1.07	0.49	1.2	3.2	1.2
**2**-6-2	738	2.6	356	7.6	0.95	0.50	1.3	2.8	1.2
**2**-6-3	979	3.2	328	7.0	0.94	0.50	1.4	2.5	1.1

a – sample name format is: starting material - reaction time (days) - replicate number, **b** – mean value for all formulae

Table S4. Summary of formula metrics for the samples analysed, considering only formulae present in all three technical replicates of each sample.

Sample[a]	Number of formulae	Shannon diversity index	*m*/*z*[b]	DBE[b]	H/C[b]	O/C[b]	Cl[b]	N[b]	F[b]
**1**-1	64	1.7	247	6.8	0.96	0.62	0.4	0.4	
**1**-3	144	1.9	290	7.0	0.92	0.61	1.3	1.8	
**1**-6	145	1.6	264	6.9	0.88	0.57	0.9	0.8	
**1**-C[c]	177	2.0	304	3.6	1.66	0.37	0.4	1.0	
**2**-1	33	0.8	293	6.9	0.89	0.45	1.0	1.4	1.2
**2**-3	42	1.1	238	5.2	1.02	0.54	1.0	1.6	0.9
**2**-6	175	1.4	260	6.2	0.80	0.52	1.2	1.3	0.9
**2**-C[c]	51	0.6	244	3.9	1.43	0.50	0.2	0.8	0.6

a – sample name format is: starting material - reaction time (days), **b** – mean value for all formulae, **c** – control sample

5. Comparison of PPL and HLB SPE cartridges

We tested both HLB and PPL cartridges on a sample (compound **2**, 3-day reaction time) to compare their extraction efficiency. The sample was prepared using the usual procedure (using 45 mg of **2**) and split into two just before extraction with either HLB or PPL cartridges. We found the extraction efficiency of PPL was 3× higher than HLB (19.3 mg *vs* 6.4 mg, respectively). Additionally, the ^{19}F NMR spectra of the two extracts show that the same major compounds are extracted with both PPL and HLB cartridges.

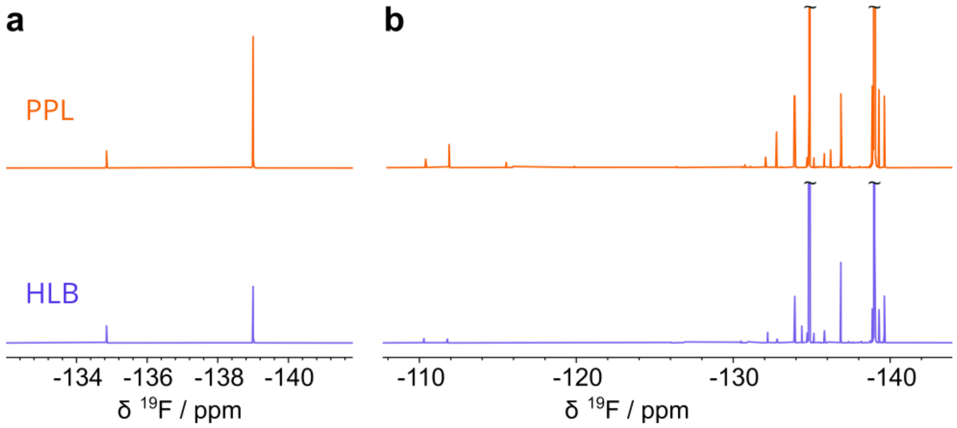

Figure S1. Comparison of 600 MHz ^{19}F {^{1}H} NMR spectra of the chloramination products of **2** after a 3-day reaction extracted using PPL and HLB SPE cartridges. Both extractions used the same amount of starting material and were dissolved in the same amount of CD_3OH. Spectrum (a) shows the starting material and the major product peaks, spectrum (b) has been vertically scaled 128-fold, to better show the comparison of the lower intensity peaks.

6. Comparison of mass spectra of 2 acquired throughout the sample workup

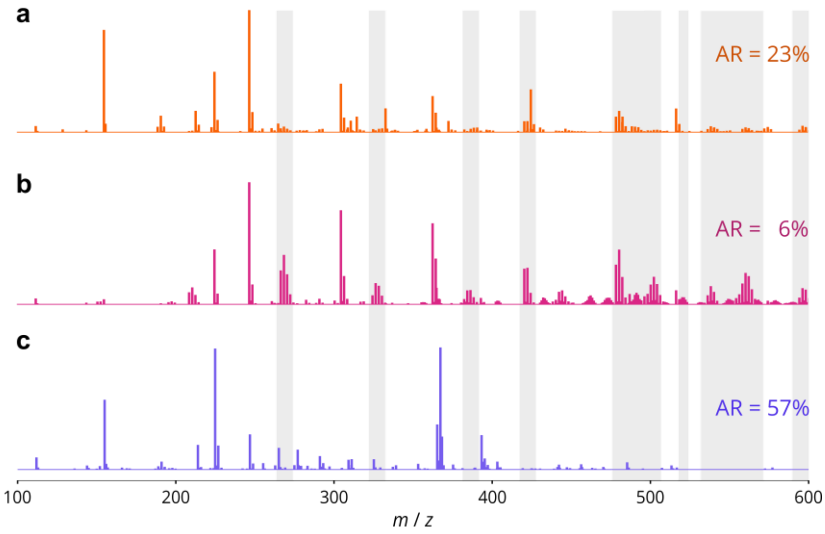

Figure S2. Mass spectra of chloramination of **2** for the day 1 sample (a) before workup, (b) SPE waste and (c) SPE eluent. Although it is possible to obtain mass spectra of the reaction mixture without sample treatment, SPE removes inorganic contaminants (highlighted in grey), which results in a higher assignment rate (AR) of the spectrum.

7. UpSet plot of 2

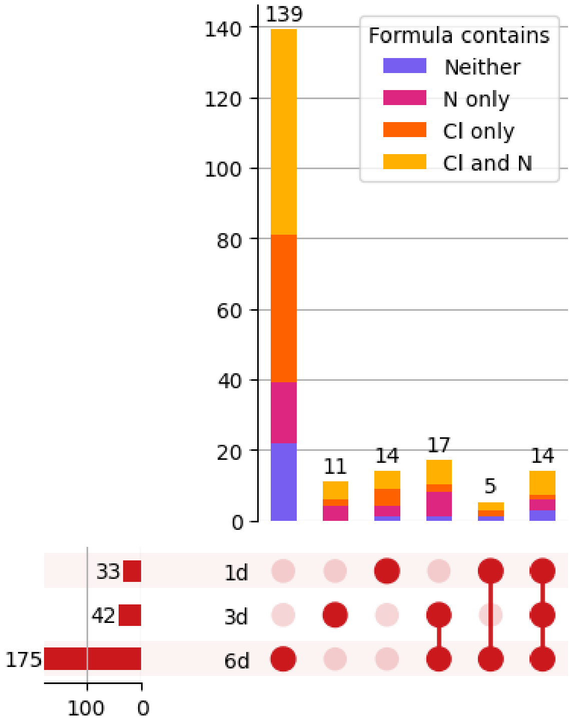

Figure S3. UpSet plot of chloramination DBPs for 1-, 3- and 6-day samples of **2**, colored by N and/or Cl content.

8. Correlation plots of 1

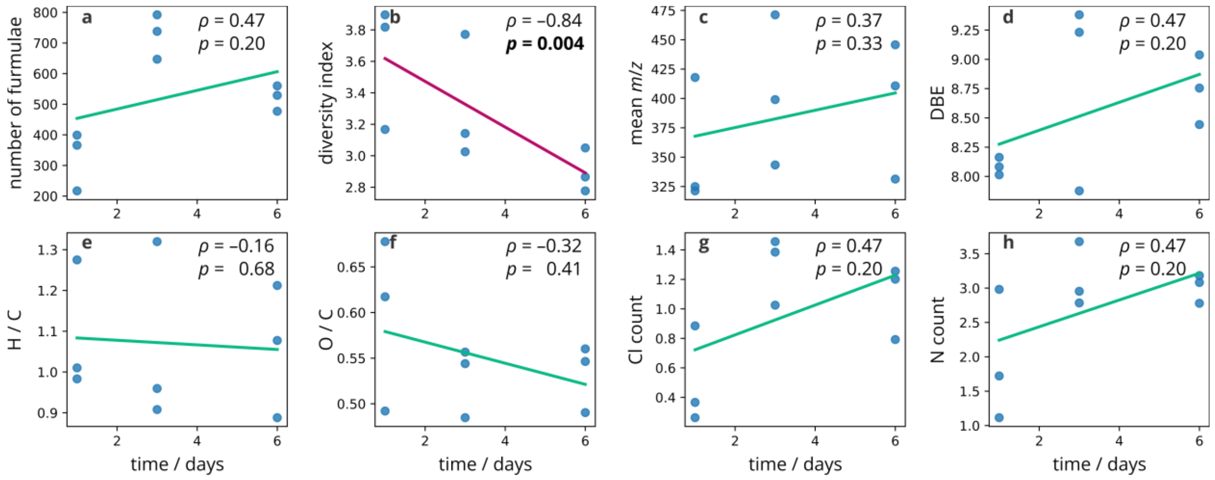

Figure S4. Correlation for chloramination of **1** of (a) average number of formulae, (b) Shannon diversity index; (c) mean m/z, (d) double bond equivalent, (e) hydrogen-to-carbon ratio, (f) oxygen-to-carbon ratio, and (g, h) count of Cl and N against time. Correlations were determined using Spearman's rho (ρ), statistically significant correlations are highlighted in red. Each point represents a technical replicate. The values of the formula metrics are shown in Table S3.

9. Comparison of weighted and unweighted Cl count means

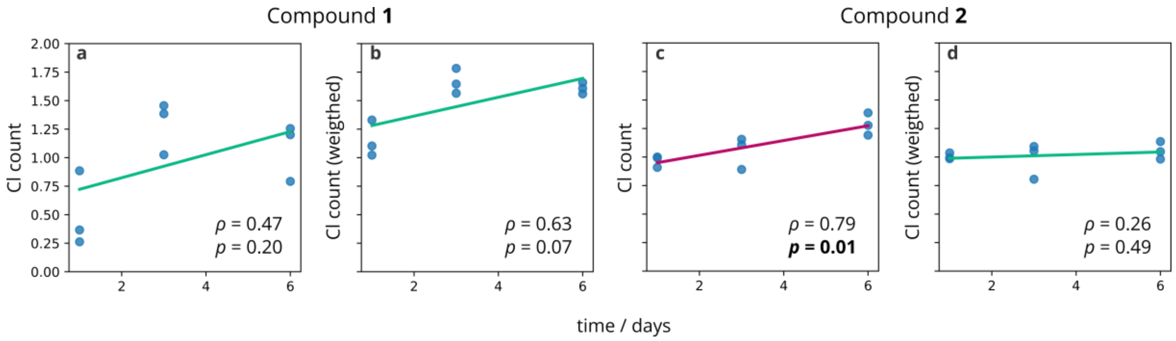

Figure S5. Correlations for chloramination of **1** *vs* time. (a) mean Cl count, (b) intensity-weighted mean Cl count (c) and (d) show equivalent data for **2**.

The use of the weighted average does not affect the significance of the correlation for **1**, however, it makes the correlation not significant for **2**. This is to be anticipated as the spectra of **2** are largely dominated by a major product containing a single Cl atom, therefore weighting by intensity results in all samples having a similar mean Cl count. Spectra of **1** on the other hand are not dominated by a single feature to such an extent (as evident from the higher Shannon diversity index) and therefore the correlation significance does not change for a weighted mean count. The one-way ANOVA test results are identical for both the weighted and unweighted Cl counts (significant difference between days for **1**, not significant for **2**).

10. Shannon index of ^{19}F NMR spectra of 2

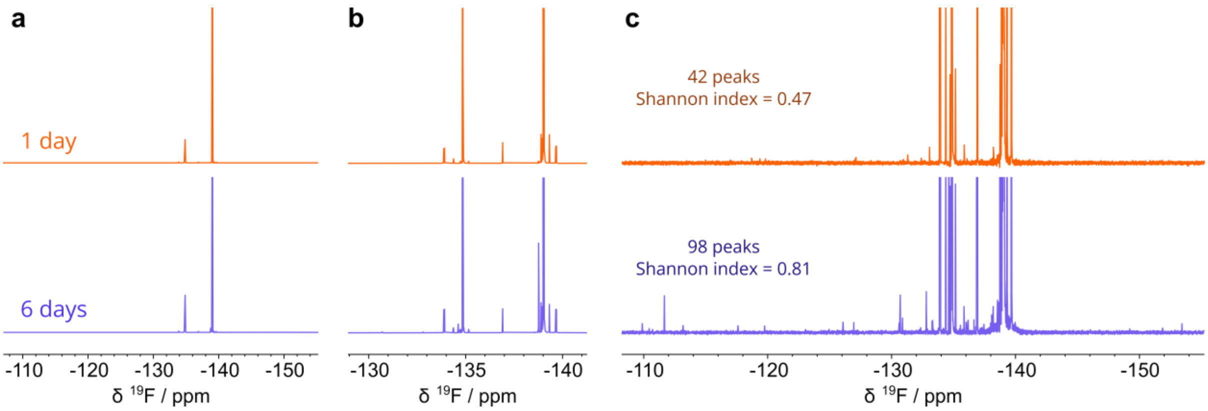

Figure S6. (a) ^{19}F {^{1}H} NMR spectra of **2** chloramination products after 1 and 6 days; and the same spectra zoomed in vertically (b) 25× and (c) 1000×. Using the Shannon index is sensible in ^{19}F {^{1}H} spectra as has been done here, as each peak is likely to come from a different compound or at least from a unique F group. A simultaneous increase in the number of peaks and the Shannon index indicates that the reaction mixture after 6 days does not become dominated by a few peaks. Note: While in theory, the Shannon index could be calculated for **1** ^{1}H spectra, this could give erroneous results, as there are likely to be multiple signals per compound (**1** has 3 signals), additionally they will be split by ^{1}H couplings, which will artificially increase the number of species and decrease their intensities, affecting the calculation of the Shannon index.

11. Isolation of high-PageRank product of 1

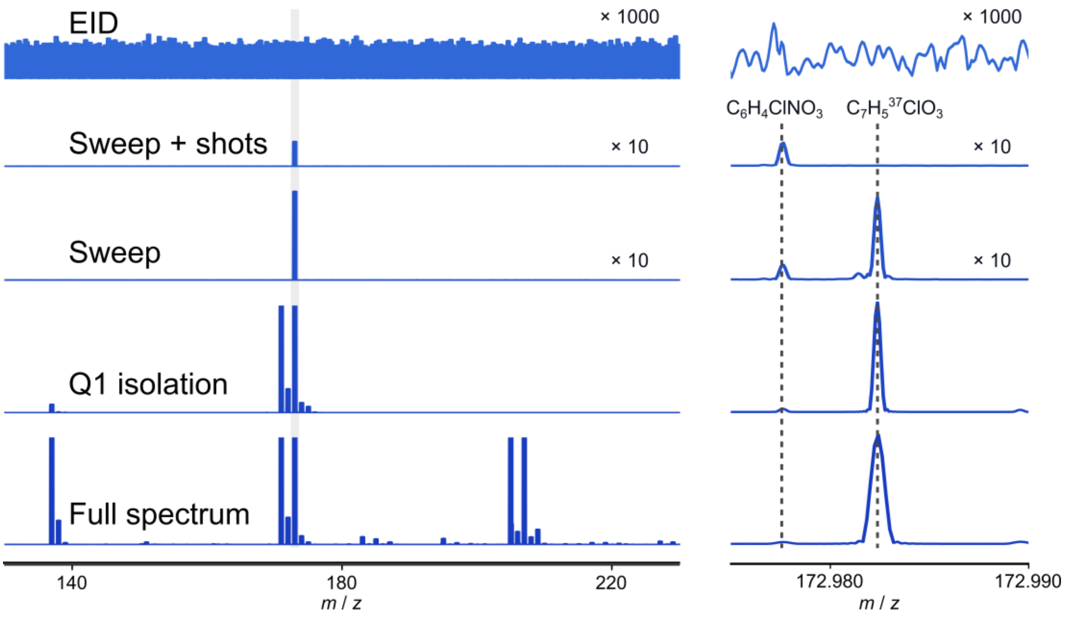

Figure S7. Isolation of high-abundance, high-PageRank product of **1**, $C_6H_4ClNO_3$ (*m/z* 172.97775). The peak could be successfully isolated from the nearest peak (4.7 mDa difference), however EID fragmentation was not successful.

12. Reaction network of 2

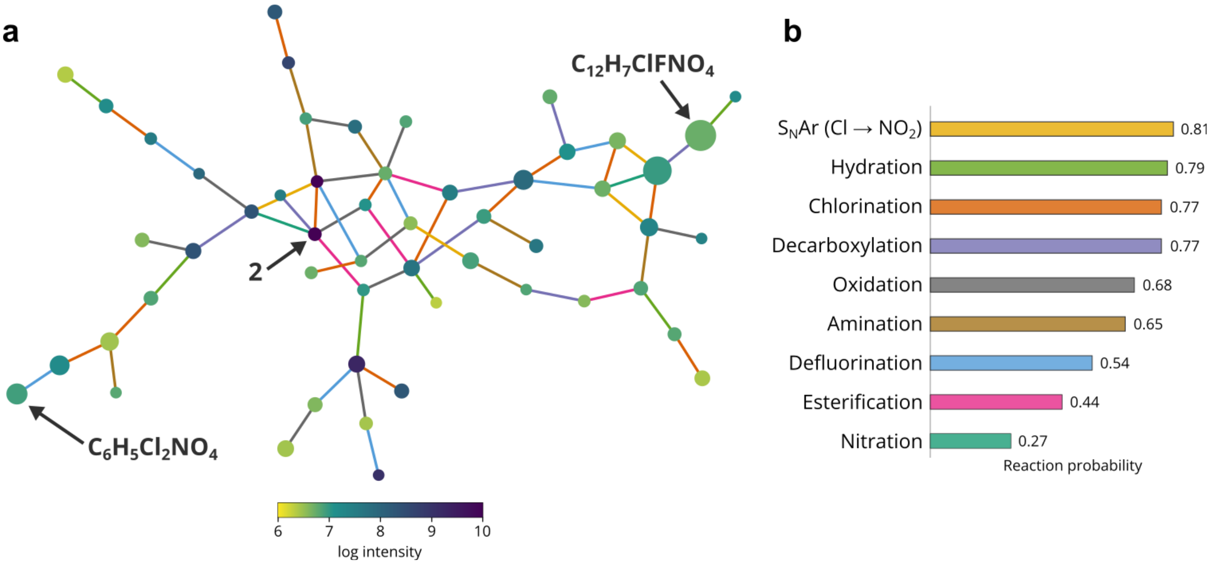

Figure S8. (a) Reaction network of chloramination DBPs of **2** after 6 days of reaction time. The nodes are scaled to their PageRank score and colored according to their MS intensity (log scale). The nodes are linked by edges which represent different reaction types. The starting material (**2**) and formulae with highest PageRank score are labelled. (b) The Reverse PageRank reaction probabilities are shown for each reaction type. S_NAr – nucleophilic aromatic substitution.

13. ^{13}C NMR analysis

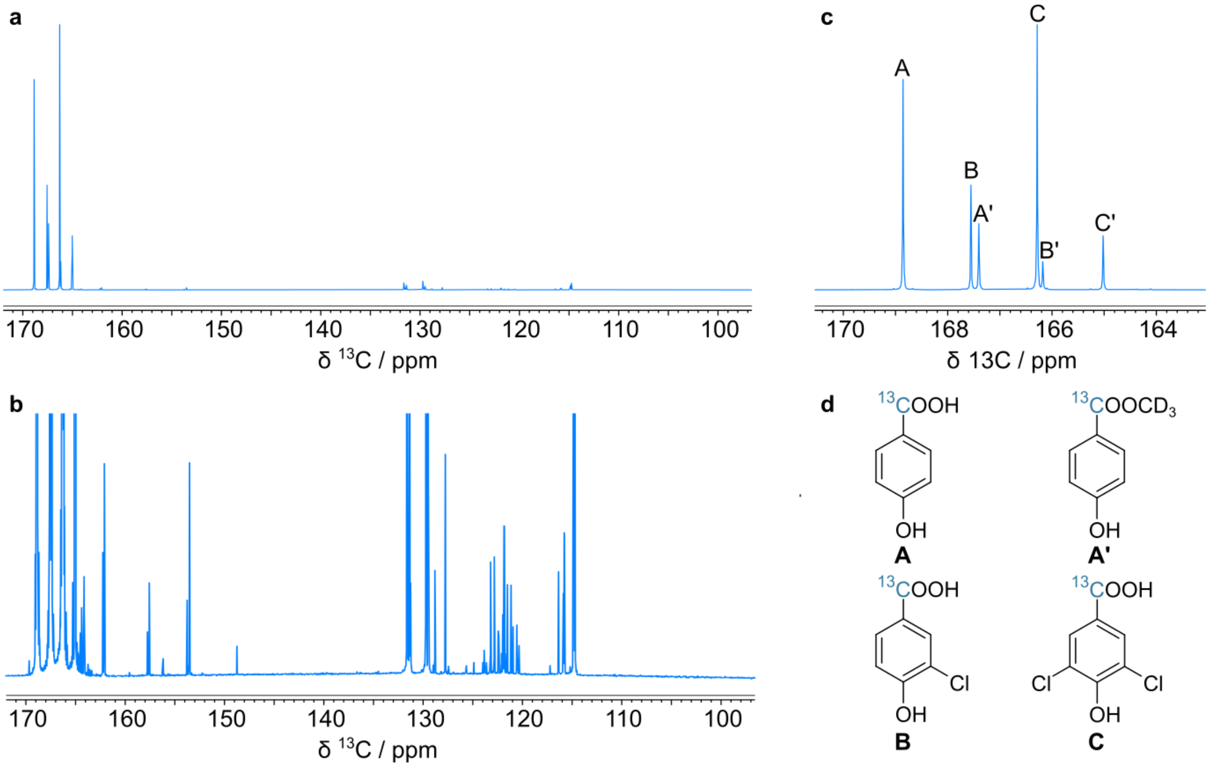

Figure S9. (a) An 800 MHz ^{13}C NMR spectrum of chloramination products of **3** after 5 days of reaction time. (b) ^{13}C NMR spectrum scaled vertically 100 times. (c) expansion of the COOH region of the spectrum, showing starting material peak (A) and the peaks of two major products (B and C). The mixture slowly reacts with the NMR solvent (CD_3OH), producing $COOCD_3$ esters (A'–C'). Their presence was confirmed by the comparing the theoretical and experimental chemical shift difference of COOH and COOMe carbonyl carbons, and by NMR and MS analysis. (d) structures of compounds A–C.

14. INADEQUATE and ADEQUATE NMR spectra

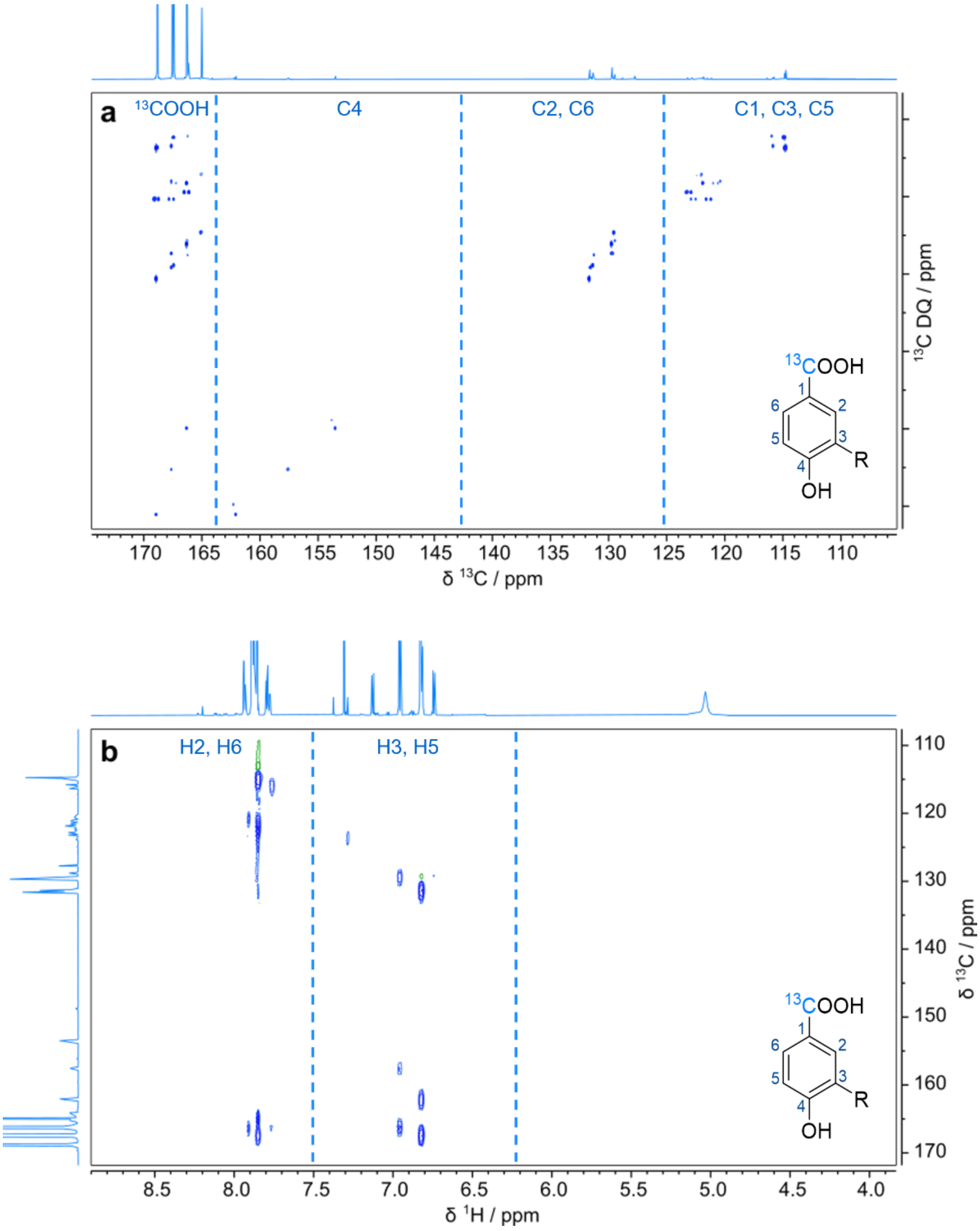

Figure S10. (a) Long-range 2D INADEQUATE and (b) 1-bond 2D ADEQUATE spectra of chloramination mixture of **3** after 5 days of reaction time.

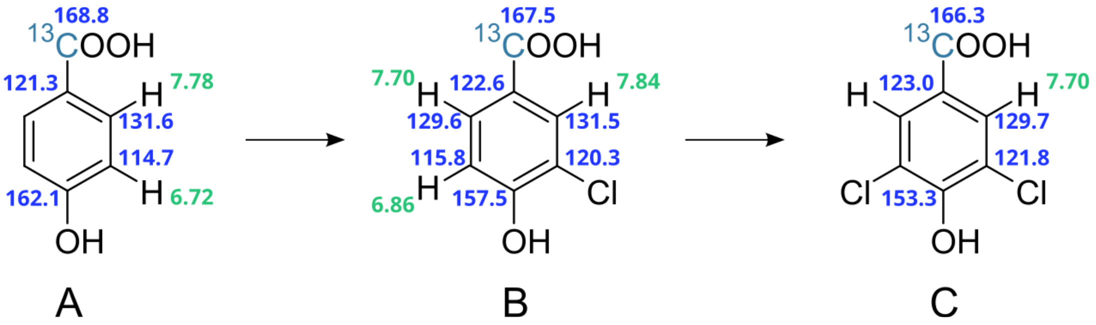

Figure S11. ^{13}C (blue) and ^{1}H (green) chemical shifts of the starting material (A) and the two major products (B and C) obtained from 2D INADEQUATE and 2D ADEQUATE spectra.

15. ^{1}H, ^{15}N HMBC spectrum of 3

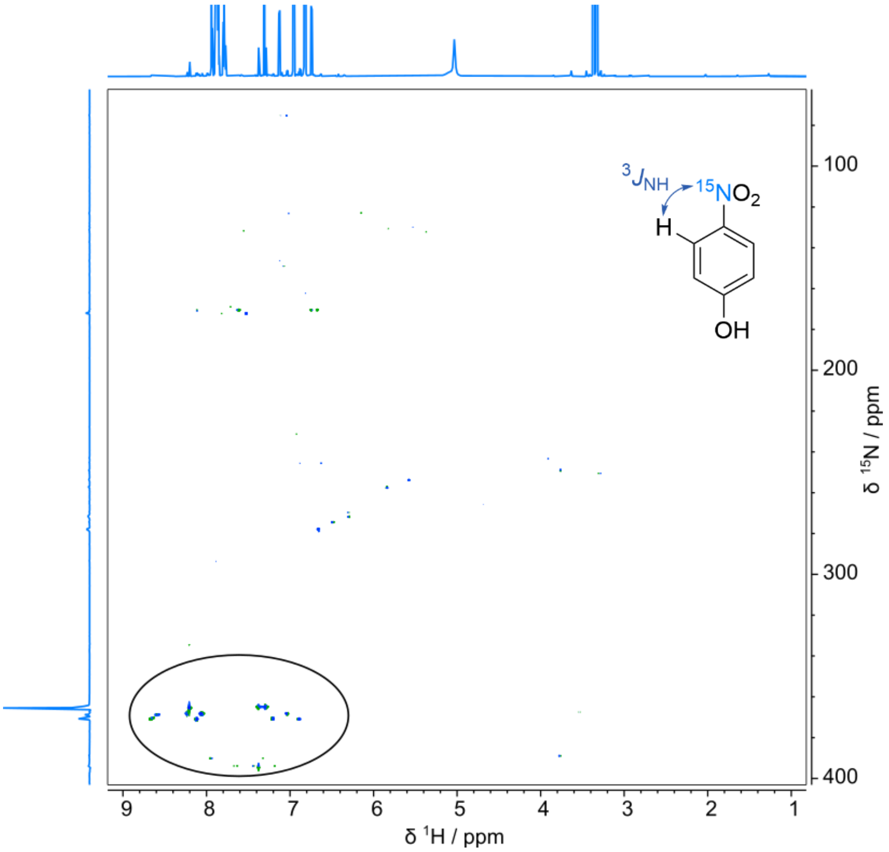

Figure S12. 2D ^{1}H, ^{15}N HMBC spectrum of chloramination mixture of **3**. The spectrum shows high intensity ^{1}H, ^{15}N correlations of nitro group peaks (circled). This agrees with the Reverse PageRank prediction of nitration being a top-rated reaction type.

References

1. Sakas, J.; Uhrín, D. More than ADEQUATE: Doubling the Sensitivity of 13CH–13CH Correlations in Double-Quantum NMR Experiments. *Chem Commun* **2022**, *58* (93), 13011–13014. https://doi.org/10.1039/D2CC05214H.
